# Follow-up study of neurodevelopment in 2-year-old infants who had suffered from neonatal hypoglycemia

**DOI:** 10.1186/s12887-019-1509-4

**Published:** 2019-04-25

**Authors:** Lin-Xia Qiao, Jian Wang, Ju-Hua Yan, Su-Xiang Xu, Hua Wang, Wen-Ying Zhu, Hai-Yan Zhang, Jie Li, Xing Feng

**Affiliations:** 1 Department of Neonatology, Children’s Hospital of Soochow University, No. 92 Zhongnan Street, Suzhou, 215025 Jiangsu China; 2 Department of Pediatrics, The First People’s Hospital, Jiangsu University, Kunshan, 215300 Jiangsu China; 3 Department of Children’s Healthcare, Kunshan Maternal and Child Health Hospital, Kunshan, 215300 Jiangsu China

**Keywords:** Neonate, Hypoglycemia, Neurodevelopment, Follow-up

## Abstract

**Background:**

Neonatal hypoglycemia is tightly related to adverse neurodevelopmental and brain injury outcomes.

**Methods:**

A total of 195 infants who were born from diabetic mothers with a low blood glucose level (< 2.6 mM) within 0.5 h after birth were enrolled in this prospective cohort study. Of these, 157 infants who had neonatal hypoglycemia (group A) were followed up, and this group was further divided into A1 [blood glucose concentration (BGC) < 2.6 mM at < 2 h after birth], A2 (BGC < 2.6 mM at 2–24 h after birth), and A3 (BGC < 2.6 mM at > 24 h after birth). A total of 144 infants whose mothers had no high risk for gestational diabetes mellitus were followed up as the control group during the same period. The neurodevelopment of the infants was evaluated by the Gesell scoring method.

**Results:**

The adaptability in the A2 and A3 subgroups was significantly lower than that in the control group (73.9 ± 6.6 vs. 87.9 ± 11.2; 71.5 ± 8.9 vs. 87.9 ± 11.2, respectively). There were significantly more mothers who used insulin during the perinatal period in A3 than in A1 and A2 (31% vs. 2%; 31% vs. 7.9%, respectively). The mothers of babies in subgroups A2 and A3 gained more weight than those of the control group (15.3 ± 1.9 kg vs. 11.1 ± 2.2 kg; 14.8 ± 2.6 kg vs. 11.1 ± 2.2 kg, respectively).

**Conclusions:**

Long and repeated neonatal hypoglycemia caused poor adaptability. The babies of mothers who used insulin or had a high weight gain during pregnancy were associated with severe or persistent neonatal hypoglycemia.

## Background

Neonatal hypoglycemia refers to the temporary condition of a decreased blood sugar level in a neonate, which is especially likely to occur in the newborns of diabetic mothers [1]. Neonates who are born from mothers with diabetes have an average rate of hypoglycemia of 8–30%, which is significantly higher than that of infants who are born from nondiabetic women (3%) [2]. With the improvement of living standards and lifestyle changes, the incidence of gestational diabetes mellitus has been increasing in recent years [3, 4]. Therefore, neonatal hypoglycemia is more frequent today than in the past. The definition of neonatal hypoglycemia is still under debate [5–8]; however, the recent guidelines from The American Academy of Pediatrics suggest that the value for the treatment of hypoglycemia is usually < 2.6 mM (45 mg/dL) after the first hours of life [9]. Other scholars have defined it as a plasma glucose level < 1.65 mM (30 mg/dL) in the first 24 h after birth [10]. Hypoglycemia is still a major metabolic abnormality in neonates [2].

A recent study has shown that neonatal hypoglycemia is tightly related to adverse neurodevelopmental and brain injury outcomes [11–14]. Glucose is an essential molecule that supplies energy for brain consumption. Neurons and glial cells in the brain are sensitive to hypoglycemia [15, 16]. An extended hypoglycemia status may induce neonatal neuroglycopenic signs, including cyanotic episodes, respiratory distress, asphyxia, bradycardia, hypothermia, and even coma and seizures [16, 17]. Consequently, maintaining glucose homeostasis is important for the general physical development of neonates [18]. The pathogenesis of hypoglycemia is very complex. Prematurity, fetal growth restriction, congenital heart disease, asphyxia, infection, islet cell hyperplasia, Beckwith-Wiedemann Syndrome, or erythroblastosis fetalis can cause neonatal stress, resulting in an imbalance of glucose homeostasis. Additionally, endocrine abnormalities including sepsis, hypothyroidism, pan-hypopituitarism, increased glucose utilization, adrenal insufficiency, and perinatal asphyxia may be related to neonatal hypoglycemia as well [2]. Nevertheless, hypoglycemia is transient and asymptomatic in most neonates; but unobserved hypoglycemia may cause neonatal nervous system injuries [19–22]. Regrettably, the effect on the neurodevelopment of the infants who had suffered from neonatal hypoglycemia still has not been clearly elucidated.

An individualized perinatal network management model of gestational diabetes mellitus was adopted in our hospital beginning in 2014. It consists of a cell phone-based app as a means to connect doctors with the parents of newborns. Thus, the parents can provide fresh data of blood glucose levels to the doctor. Both systematic blood glucose monitoring and intervention of newborns after birth are included in the network management model. In this study, we aimed to follow up the neurodevelopment of infants who had suffered from neonatal hypoglycemia and to determine whether this monitoring and intervention system in neonatal hypoglycemia is appropriate.

## Methods

### Patients

**Fig. 1:**
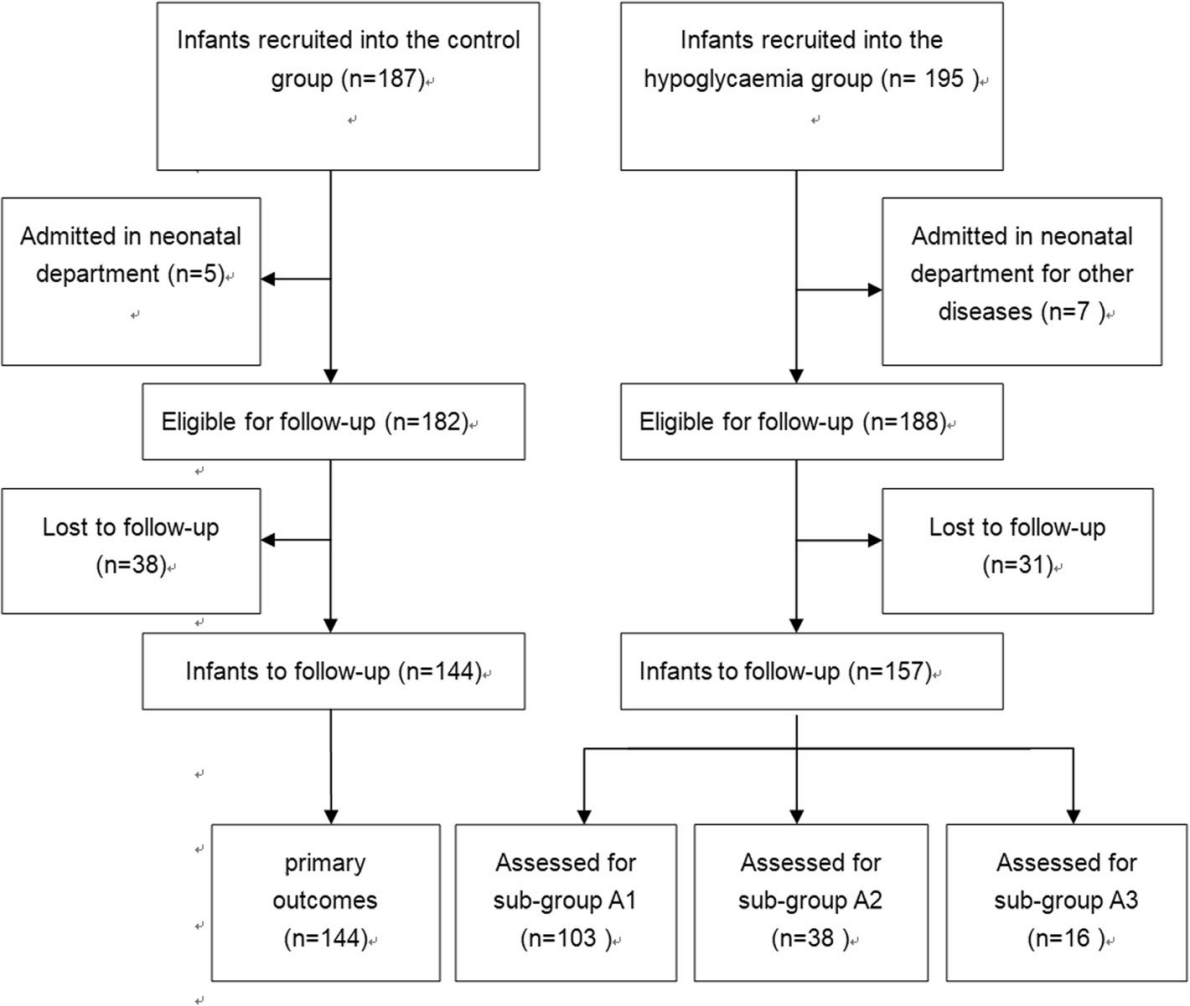
Flowchart showing the process for selection of eligible infants in this study. n is the number of cases

A total of 195 infants who were born from diabetic mothers with a low blood glucose level (< 2.6 mM) within 0.5 h after birth were enrolled in this prospective cohort study. Those infants whose mother used insulin were excluded in this study. Among these infants, five babies were transferred to the Neonatal Department because of other disorders. Thus, 190 babies underwent the investigation process during the neonatal period in group A. A total of 187 normal, full-term infants whose mother had no high risk for gestational diabetes mellitus were preliminarily recruited into the control group during the same period. Among them, five babies were excluded because of another disease. Finally, 182 babies continued forward into the next observation in the control group (Fig. 1). The infants who had either neonatal asphyxia or brain dysplasia were excluded from this study. All infants enrolled in this study were born at the First People’s Hospital in Kunshan, Jiangsu University. All parents of the infants agreed and signed a consent for this study.

### Neonatal hypoglycemia

Hypoglycemia was defined as at least one episode of blood glucose concentration (BGC) less than 2.6 mM [23].

### Treatment and groups

Neonates with hypoglycemia formed group A. Full-term babies of normal health belonged to the control group. The neonates in group A were treated with additional feeding, intravenous dextrose, or buccal dextrose gel to maintain a BGC ≥ 2.6 mM. Briefly, 2 mL/kg 10% glucose was orally administered to the hypoglycemic babies (group A), and, 30 min later, the BGC was measured again. If the BGC was still < 2.6 mM, an additional dose of 2 mL/kg 10% glucose was given. Another 30 min later, if the BGC was > 2.6 mM, the baby would stay with his/her mom with continuous monitoring of the BGC. The babies whose GBC was still < 2.6 mM were transferred to the Neonatal Department and administered a glucose supplement, either orally or intravenously, until the BGC was > 2.6 mM. For subclassification, once the BGC recovered to > 2.6 mM within 2 h after birth, the babies were divided into subgroup A1. If the BGC recovered to > 2.6 mM at the time of 2–24 h after birth, the babies were divided into subgroup A2. If the BGC was still < 2.6 mM at more than 24 h after birth, the babies were divided into subgroup A3. If the BGC of the babies in subgroup A1 or subgroup A2 decreased below 2.6 mM after 24 h of birth, they were divided into subgroup A3.

### Ethics authorization

The studies both during the neonatal period and at the 2-year follow-up were authorized by the Ethics Committee of the First People’s Hospital of Kunshan. A written informed consent was signed by the parents of the infants at study entry.

### BGC measurement

A microglucose meter, which was equilibrated before use each time, and the corresponding test paper (Roche, Basel, Switzerland) were employed to measure the BGC. A sample of 1–2 drops of capillary blood from the baby’s heel was taken and dropped directly onto the test paper to fully cover it within 1 min. Then, the test paper was inserted into the microglucose meter. The measured BGC level was displayed automatically.

### Follow-up

All the surviving infants enrolled in this study were followed up at a corrected age of 2 years old. In groups A and B, 31 and 38 cases were lost, respectively; therefore, 157 and 144 infants underwent the follow-up evaluation, respectively. In group A, 103 infants were in group A1, 38 infants were in group A2, and 16 infants were in group A3 (Fig. 1). The neurodevelopment of the infants was followed up between January 2016 and November 2016 at the Kunshan Maternal and Child Health Hospital. The infants were evaluated independently by two assessors, who were blinded to the infant information and neonatal blood sugar status, and their scores were calculated based on the average of the two evaluations.

### Neurodevelopment evaluation by the Gesell scoring method

The neurodevelopment of the infants was evaluated by the Gesell developmental test (Chinese revised version), which was performed by an experienced and professional Doctor of Child Health Care. According to Gesell’s scoring method, five energy areas of children’s actions were analyzed, including physical activity, speech energy, and human energy [24]. Independent doctors were employed to perform the Gesell Infant Development Scale (GESELL) (Chinese revised edition) to measure five parameters: gross motor skills, fine motor skills, adaptability, language, and personal social activity. The observed behavior pattern was compared to the corresponding normal behavior. The infant development score was calculated according to the following formula: DQ = DA/CA × 100, where DA = (sigma (M × N) (N) / sigma), DQ is the developmental quotient, DA is the childbearing age, CA is the actual age, M is the infant age in months divided by the score, and N is the number of the positive signals in the monthly items. Infant development was defined as follows: DQ <  70 is abnormal, DQ = 70–84 is suspected abnormal, and DQ > 85 is normal. During treatment, DQ served as an indicator for the degree of development disorder (DD).

### Profile of the infants on follow-up

A total of 195 infants were enrolled in group A. Among them, five babies were transferred to the Neonatal Department because of other disorders. Thus, 190 babies underwent the investigation process during the neonatal period. At the time of follow-up, 31 cases were lost. Finally, 157 infants in group A underwent the follow-up evaluation. Among them, 103 infants were in group A1, 38 infants were in group A2, and 16 infants were in group A3. As normal controls, 187 infants were preliminarily recruited (control group) during the same period. Among them, five babies were excluded because of another disease. At the time of follow-up, 38 cases were lost; thus, 144 infants in the control group underwent the follow-up evaluation.

### Statistical analysis

All data were analyzed by using SPSS 21.0 software. The results were presented as the mean ± standard deviation (SD) (x ± s), median, or interquartile range. Differences in rates were analyzed by the chi-squared test or the T test. *P* < 0.05 was considered a statistically significant difference.

## Results

### General characteristics of the study subjects

A total of 382 babies were preliminarily recruited into the study (195 in the hypoglycemia group (group A) and 187 in the control group). The follow-up observation was carried out when the infants reached 2 years old (corrected age ± 2 months). Ultimately, 301 infants underwent the study (144 in the control group and 157 in the hypoglycemia group) (Fig. 1). Their mean (SD) age at the time of neurodevelopmental assessment was 2.0 (0.1) years, and 162 (54%) were male. There were no significant differences in sex, gestation, birth weight, Apgar score, cases of breast feeding, cases of ventilation, or cases of feeding influence between the hypoglycemia and the control groups. The adaptability in the A2 and A3 subgroups was significantly lower than that in the controls (73.9 ± 6.6 vs. 87.9 ± 11.2; 71.5 ± 8.9 vs. 87.9 ± 11.2, respectively). Significantly more mothers used insulin in the perinatal period in subgroup A3 than in the other two subgroups (31% vs. 2%, *p* < 0.0001, 31% vs. 7.9%, *p* = 0.027, respectively). The weight gain of the mother during pregnancy in subgroups A2 and A3 was significantly higher than that in the control group (15.3 ± 1.9 kg vs. 11.1 ± 2.2 kg, *p* = 0.0154; 14.8 ± 2.6 kg vs. 11.1 ± 2.2 kg, *p* = 0.0342, respectively); however, no significant difference was found between subgroup A1 and the control group (112.4 ± 3.5 kg vs. 11.1 ± 2.2 kg, *p* = 0.7452) (Table 1).

**Table 1:** General characteristics of the infants in this study

Group	Hypoglycemia group (Group A)			Control group
Subgroup	Group A1	Group A2	Group A3	
n	103	38	16	144
Male (%)	54 (52.4)	22 (55.3)	9 (56)	77 (53.5)
Gestation (weeks)	37.8 ± 1.4	38.2 ± 0.8	37.6 ± 1.1	38.9 ± 0.7
Birth weight (g)	3223 ± 347	3468 ± 365	3542 ± 432	3384 ± 242
Apgar score (5 min)	8.7 ± 0.3	9.1 ± 0.4	8.9 ± 0.3	9.1 ± 0.5
Cases of ventilation (%)	4 (3.9)	0	1 (6.3)	3 (2.1)
Cases of feeding intolerance (%)	6 (5.8)	3 (7.9)	1 (18.8)	7 (4.9)
maternal insulin (%)	2 (2.0)	3 (7.9)^△^	5 (31.0) ^# △^	0 (0)
W-gain. Preg-mother (kg)	12.4 ± 3.5	14.8 ± 2.6^△^	15.3 ± 1.9^△^	11.1 ± 2.2
Cases of breast feeding (%) (at 2 weeks) (total volume > 50%)	84 (81.2)	24 (63.2)	9 (56.3)	123 (85.4)

*t test, # chi-squared test; ^△^ *p* < 0.05, compared with the control group. W-gain. Preg-mother: weight gain of the mother in pregnancy

### Neurodevelopmental indices

The evaluation of the neurodevelopment at a corrected age of 2 years old of the infants showed no significant difference in any assessment score of neurodevelopment (including gross motor, fine motor, adaptability, language, and social skills) between group A and the control group. The adaptability scores of subgroups A2 and A3 were significantly lower than that of the control group (73.9 ± 6.6 vs. 87.9 ± 11.2, *p* = 0.0243; 71.5 ± 8.9 vs. 87.9 ± 11.2, *p* = 0.0138, respectively). In addition, the adaptability score was not significantly different between subgroup A1 and the control group. Moreover, other scores of neurodevelopment (including gross motor, fine motor, language, and social skills) were not significantly different among the subgroups and the control group (Table 2).

**Table 2:** Infant neurodevelopment assessment at 2 years old

Group	Hypoglycemia group (Group A)			Control group
	Group A1	GroupA2	Group A3	
n	103	38	16	144
Gross motor	87.4 ± 10.3	84.6 ± 7.8	83.5 ± 11.4	86.1 ± 9.4
Fine motor	90.2 ± 6.2	86.8 ± 8.2	85.4 ± 9.6	91.8 ± 12.9
Language	91.7 ± 12.3	88.5 ± 10.3	89.3 ± 8.7	89.6 ± 11.4
Adaptability	83.6 ± 11.9	73.9 ± 6.6^△^	71.5 ± 8.9^△^	87.9 ± 11.2
Social skills	84.4 ± 11.4	82.6 ± 6.9	80.5 ± 9.8	84.8 ± 13.6

^△^ *p* < 0.05

## Discussion

In this study, 144 infants who had neonatal hypoglycemia were analyzed for their neurodevelopment by the Gesell scoring method to investigate their gross motor, fine motor, adaptability (including the abilities of fine-motor coordination for objects and scenes, hand-eye coordination, problem solving, and application tools), language, and social skills at 2 years old. We found that long and repeated neonatal hypoglycemia, especially that lasting for more than 24 h, affected neurodevelopment and was associated with a high risk of poor adaptability. Indeed, studies in newborns with hypoglycemia by magnetic resonance imaging have shown that edema occurs in the posterior occipital and cortex region, with symmetrical changes [25, 26]. The occipital and cortex regions are somatosensory and visual control areas [27], which impact cognitive skills, adaptability, and visual skills. Under hypoglycemic conditions, the liver glycogen reserves are insufficient. Once the blood sugar level reaches the lowest point, the synthesis of lipids, proteins, DNA, and RNA is limited or delayed because not enough energy is lied, thus affecting brain cell metabolism and development and eventually leading to neuronal necrosis. A high level of glucose is required for the occipital region because there are more neurons and synapses in this region [28]. If hypoglycemia is not able to be quickly corrected, irreversible brain damage in the posterior occipital and cortex regions will result.

Neonatal hypoglycemia is a common metabolic disorder during the neonatal period. Volpe has indicated that continuous, repeated hypoglycemia can cause brain damage [29]. In addition, Filan et al. have found that neonatal hypoglycemia can injure the occipital brain, resulting in long-term disability, visual impairment, and epilepsy [28]. In this study, at a corrected age of 2 years old, no significant difference was found in any assessment score of neurodevelopment (including gross motor, fine motor, adaptability, language, and social skills) between the infants who had neonatal hypoglycemia and controls. This result seems similar with that of Christopher et al., who found that neonatal hypoglycemia is not related to adverse neurodevelopment at 2 years old [30]. However, since neonatal hypoglycemia occurred at different times in group A, we further divided this group into A1 (neonatal hypoglycemia within 2 h of birth), A2 (neonatal hypoglycemia at 2–24 h of birth), and A3 (neonatal hypoglycemia at more than 24 h of birth). Interestingly, the adaptability scores in subgroups A2 and A3 were significantly lower than that of the control group (73.9 ± 6.6 vs. 87.9 ± 11.2, *p* = 0.0243; 71.5 ± 8.9 vs. 87.9 ± 11.2, *p* = 0.0138, respectively). This finding indicated that temporary hypoglycemia (within 2 h) did not induce neurodevelopmental injury. However, long and repeated hypoglycemia decreased adaptability development. In a follow-up study at 4.5 years old, neonatal hypoglycemia was found to increase the risk of poor executive function, visual skills, and fine motor skills [2], especially in infants with hypoglycemia at more than 24 h after birth. This result is different from our findings. The reason might be because of the different observation age and assessment methods. Thus, further follow-up after a longer time period and with different methods is necessary. The etiology of adverse neurodevelopment caused by neonatal hypoglycemia is unclear. Filan et al. believe that transient hyperinsulinism is an independent risk factor for neonatal hypoglycemia [28]. Thus, an animal model study would be valuable. There is no clear consensus on the management of neonatal hypoglycemia. Over or under supplementation with sugar would potentially damage the brain [9, 31]. Therefore, how to balance the risks is still a challenge [32]. Among our results, there were no significant differences in sex, gestation, birth weight, Apgar score, or cases of breast feeding between the neonatal hypoglycemia group and the normal control group. However, significantly more mothers used insulin during the perinatal period in subgroup A3 than in subgroups A2 and A3 (31% vs. 2%, *p* < 0.0001; 31% vs. 7.9%, *p* = 0.027, respectively). In addition, the weight gain of the mother during pregnancy in subgroups A2 and A3 was significantly higher than that in the control group (15.3 ± 1.9 kg vs. 11.1 ± 2.2 kg, *p* = 0.0154; 14.8 ± 2.6 kg vs. 11.1 ± 2.2 kg, *p* = 0.0342, respectively). No obvious difference between subgroup A1 and the control group was found (112.4 ± 3.5 kg vs. 11.1 ± 2.2 kg, *p* = 0.7452).

The limitations of this study include a small sample size for the subgroups, the study being conducted in a single center, and a short follow-up time of 2 years. Therefore, observation of the long-term effects of neonatal hypoglycemia is necessary because fine motor development of children occurs until they reach 8 years old.

## Conclusions

This study indicates that the babies whose mother had gained more weight or used insulin during the perinatal period have a higher possibility of long and repeated neonatal hypoglycemia. Thus, pediatricians should pay more attention to the babies whose mother had a history of insulin use or a high weight gain.

## Abbreviations

BGC: Blood glucose concentration
